# NRF2, a Key Regulator of Antioxidants with Two Faces towards Cancer

**DOI:** 10.1155/2016/2746457

**Published:** 2016-06-02

**Authors:** Jaieun Kim, Young-Sam Keum

**Affiliations:** ^1^Department of Pathology, College of Korean Medicine, Dongguk University, Donggukro 32, Goyang, Gyeonggi-do 10326, Republic of Korea; ^2^College of Pharmacy, Dongguk University, Donggukro 32, Goyang, Gyeonggi-do 10326, Republic of Korea

## Abstract

While reactive oxygen species (ROS) is generally considered harmful, a relevant amount of ROS is necessary for a number of cellular functions, including the intracellular signal transduction. In order to deal with an excessive amount of ROS, organisms are equipped with a sufficient amount of antioxidants together with NF-E2-related factor-2 (NRF2), a transcription factor that plays a key role in the protection of organisms against environmental or intracellular stresses. While the NRF2 activity has been generally viewed as beneficial to preserve the integrity of organisms, recent studies have demonstrated that cancer cells hijack the NRF2 activity to survive under the oxidative stress and, therefore, a close check must be kept on the NRF2 activity in cancer. In the present review, we briefly highlight important progresses in understanding the molecular mechanism, structure, and function of KEAP1 and NRF2 interaction. In addition, we provide general perspectives that justify conflicting views on the NRF2 activity in cancer.

## 1. Introduction

A growing body of evidence indicates that oxidative stress is responsible for the development of chronic diseases, such as cancer, diabetes, atherosclerosis, neurodegeneration, and aging [1, 2]. Oxidative stress results from a perturbation between the production and removal of reactive oxygen species (ROS). ROS refers to free radical and non-free-radical oxygenated molecules, such as superoxide (O_2_^−^), hydrogen peroxide (H_2_O_2_), and hydroxyl radical (OH^−^). The majority of exogenous ROS is generated in organisms after exposure to oxidants and electrophiles, such as pollutants, tobacco, smoke, drugs, and xenobiotics [3]. Ionizing radiation also generates ROS through the direct activation of water, a process termed radiolysis [4]. On the other hand, intracellular ROS can be generated from many sources: cytosolic NAPDH oxidases (NOXs) take part in the regulated generation of ROS, while ROS is generated as by-product of the oxidative phosphorylation in mitochondria [5, 6]. Other significant sources of cellular ROS production include xanthine oxidase [7]. Oxidative metabolic process in peroxisomes cannot be negligible as well [8]. It is known that low levels of intracellular ROS are necessary to carry out a number of important physiological functions, such as intracellular signal transduction and host defense against microorganisms. However, high levels of intracellular ROS are considered detrimental because they impart significant oxidative damage on cellular macromolecules, such as nucleotides, lipid, and proteins [9].

In order to fight against the oxidative stress, organisms create a highly reducing intracellular environment by maintaining a large amount of antioxidant molecules, such as reduced glutathione (GSH) and soluble vitamins (vitamin C and vitamin E) [10, 11]. During evolution, organisms have also developed a variety of cellular defensive enzymes, such as alcohol dehydrogenase and aldehyde dehydrogenase to ATP binding cassette (ABC) transporters that mediate the adaptive responses to survive under the oxidative environment and xenobiotic assault. The first defense metabolism, for example, phase I reaction, is carried out by cytochrome P450 enzymes that catalyze the monooxygenation reaction of substrates [12], for example, the insertion of one atom of oxygen into the aliphatic position of an organic substrate with the other oxygen atom reduced to water. A group of enzymes, including uridine 5′-diphospho-glucuronosyltransferases (UGT), glutathione S-transferases (GST), or sulfotransferases, carry out the subsequent reaction, referred to as phase II reaction, in which the hydroxylated metabolites are further conjugated with soluble molecules, such as glutathione, sulfate, glycine, and glucuronic acid [13]. Finally, the addition of these large anionic groups produces metabolites completely soluble in cells, which can be actively transported out, a process referred to as phase III reaction [14].

## 2. The Triad of ROS: Superoxide (O_2_^−^), Hydrogen Peroxide (H_**2**_O_**2**_), and Hydroxyl Radical (OH^−^) and Their Biological Targets for Signaling

The first type of ROS, superoxide (O_2_^−^), is generated by the one-electron reduction of O_2_ through the electron transport chain in mitochondria. Superoxide can also be produced by a family of NADPH oxidases (NOXs), using oxygen and NADPH as substrates [15], in which superoxide is rapidly disposed. The second type of ROS, hydrogen peroxide (H_2_O_2_), is rapidly formed in the cytoplasm, from O_2_^−^ by superoxide dismutase 1 (SOD1), while extracellular SOD (SOD3) produces H_2_O_2_ outside the cell. Superoxide produced in the matrix of mitochondria is converted into H_2_O_2_ by superoxide dismutase 2 (SOD2) [16]. In addition, H_2_O_2_ can be produced as a by-product during *β*-oxidation of fatty acids in the peroxisome or by a wide array of cellular enzymes, including cytochrome P450s [17]. Finally, H_2_O_2_ is converted into harmless water and O_2_ by various cellular antioxidant enzymes, such as peroxiredoxins (PRXs), glutathione peroxidases (GPXs), and catalases (CAT). While PRXs and GPXs are present in most cell compartments, catalase is confined to the peroxisome. In addition, PRXs are among the most abundant enzymes and have been suspected of degrading most of hydrogen peroxide with a slow rate whereas GPXs seem to be less abundant but have higher rate constants [18].

It is noteworthy that H_2_O_2_ is a bona fide signaling molecule. H_2_O_2_ is stable and readily diffuses across the membrane, thereby oxidizing cysteine residues of redox-sensitive proteins. Susceptible cysteine residues in redox-sensitive proteins exist as a thiolate anion in a physiological pH and they can be reversibly oxidized by hydrogen peroxide to yield sulfenic acid (SO^−^). When hydrogen peroxide level is sufficiently high, sulfenic acid can undergo a further hyperoxidation into sulfinic (SO^2−^) and sulfonic (SO^3−^) acids, in which the formation of sulfonic acid is considered as an irreversible oxidative modification [19, 20]. Alternatively, the Fenton reaction can produce the third type of ROS, hydroxyl radical (OH^−^), from H_2_O_2_ by accepting an electron from free cations (Fe^2+^ or Cu^+^). Although recent studies provide some evidence that O_2_^−^ and OH^−^ can participate in transmitting the signal transduction, the detailed molecular mechanisms are still largely unclear [21]. Together, it is likely that the type and local concentration of ROS determine whether redox signaling is transmitted or the oxidative-stress induced damage occurs in cells.

Mitogens, such as epidermal growth factor (EGF) and platelet-derived growth factor (PDGF), promote the rate of cell growth and proliferation by activating membrane-bound receptor tyrosine kinases (RTKs) via the autophosphorylation of specific tyrosine residues on the cytoplasmic tails [22]. This event results in the recruitment of multiple adaptors to RTKs and promotes subsequent activation of downstream signal transduction cascades. On the other hand, protein tyrosine phosphatases (PTPs) carry out tyrosine dephosphorylation of these receptors, thereby switching off the signal transduction cascades [23]. Interestingly, previous studies have demonstrated that the oxidation of catalytic cysteine residues in PTPs contributes to the inactivation and sustained promotion of cell growth and proliferation. For example, EGF treatment can generate intracellular H_2_O_2_ and promote the inactivation of protein tyrosine phosphatase 1B (PTP1B) by oxidizing the catalytic cysteine residues into sulfenic acid [24]. Likewise, PDGF treatment led to the generation of intracellular H_2_O_2_ and caused oxidation of cysteine residues of the PDGFR-associated phosphatase, SHP-2 [25]. Moreover, H_2_O_2_ can promote the cysteine oxidation of PTEN, a PTP that removes the phosphate from phosphatidylinositol and serves as a critical regulatory molecule of PI3K/Akt signaling cascade [26, 27]. Together, these results suggest that oxidizing cysteine residues in PTPs by H_2_O_2_ is an important switch to assist in the cell growth or proliferation by growth factors. In addition, it is also possible to speculate that the oxidation of cysteine residues in unknown redox-sensitive proteins other than PTPs might contribute to the signal transduction by hydrogen peroxide.

## 3. Structural Insights into NRF2 and KEAP1 Regulation

**Figure 1:**
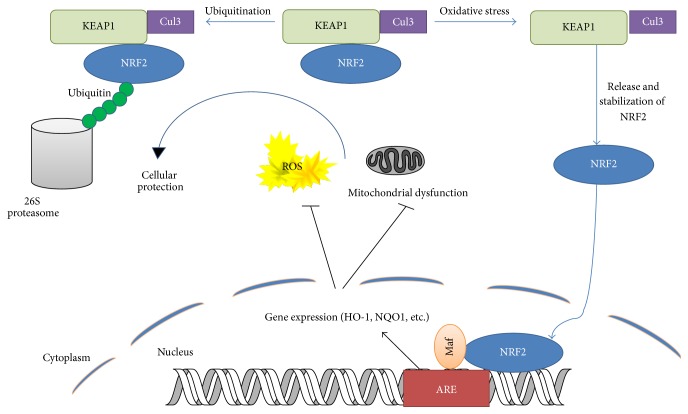
Regulation of NRF2 stability by KEAP1. NRF2 is constantly degraded by KEAP1-mediated ubiquitination in the cytoplasm. Oxidative stress will halt degradation of NRF2 and lead it to bind to ARE to activate transcription of oxidant and detoxifying enzymes.

While a moderate amount of ROS can affect the cellular signaling activity by modifying cysteine residues in redox-sensitive proteins, an excessive amount of ROS is toxic and must be eradicated. The removal of intracellular ROS is carried out, at least in part, by a number of phase II cytoprotective enzymes, including heme oxygenase-1 (HO-1), NAD[P]H:quinone oxidoreductase-1 (NQO1), glutathione S-transferase (GST), and *γ*-glutamylcysteine ligase (*γ*-GCS) (Figure 1) [28, 29]. It is widely accepted that transcription of these enzymes is regulated by the antioxidant response element (ARE), a* cis*-acting DNA sequence that exists in the 5′-upstream promoter of these genes [30, 31]. NF-E2-related factor-2 (NRF2) is a transcriptional factor that binds to and mediates the ARE-dependent gene activation. Under a basal condition, NRF2 is sequestered in the cytoplasm and its expression is maintained to be low due to constant polyubiquitination. In response to a variety of stresses, NRF2 is significantly induced and translocates into the nucleus, where it activates the ARE-dependent gene expression in association with small Maf proteins and other coactivators. Detailed domain analyses have revealed that NRF2 comprises six conserved NRF2-ECH (Neh) domains. The Neh1 domain contains a basic leucine zipper motif (bZIP) and behaves as a platform for binding to the ARE. The Neh2 domain is located in the most N-terminal region and acts as a negative regulatory domain. The Neh3 domain is located in the most C-terminal region and plays a permissive role for NRF2 transactivation. The Neh4 and Neh5 domains seem to be essential for NRF2 transactivation and the Neh6 domain is required for NRF2 protein degradation [32, 33]. However, the detailed studies elucidating the in-depth function of individual domains of NRF2 are not available and required to fully characterize the exact molecular functions of individual domains.

**Figure 2:**
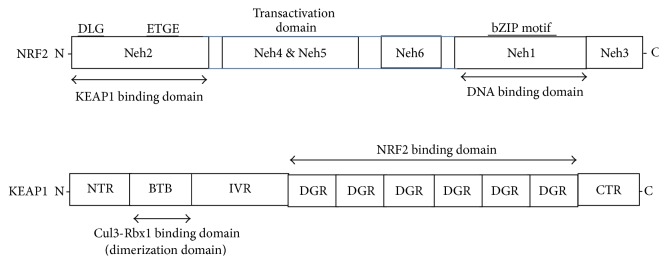
Domain structure of NRF2 and KEAP1 proteins. bZIP: basic leucine zipper, NTR: N-terminal region, BTB: Broad Complex, Tramtrack, and Bric-a-Brac, IVR: intervening region, DGR: double glycine repeat (=Kelch), and CTR: carboxyl terminal region.

**Figure 3:**
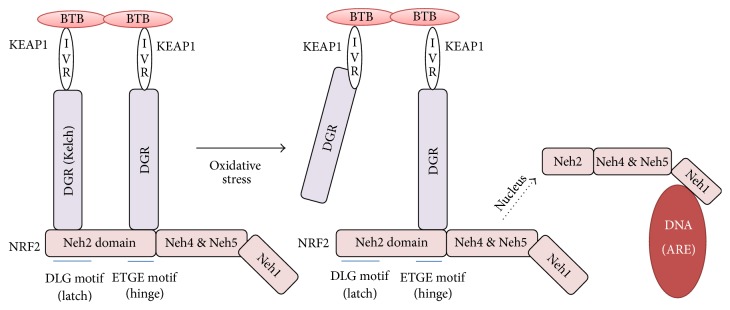
Interaction of NRF2 and KEAP1: hinge and latch model. KEAP1 proteins dimerize via BTB domains. The KEAP1 homodimer identifies the DLG (weak interaction) and ETGE (strong interaction) motifs in the NRF2. NRF2 tightly binds to KEAP1 homodimer in basal state. After stress, weaker DLG motif is detached, blocking ubiquitination of NRF2 and facilitating nuclear import and binding to ARE. BTB: Broad Complex, Tramtrack, and Bric-a-Brac, IVR: intervening region, DGR: double glycine repeat (=Kelch), and ARE: antioxidant response element.

Kelch-like ECH-associated protein 1 (KEAP1) was initially identified by yeast 2-hybrid assay as a novel binding partner of NRF2, using the Neh2 domain as bait [34]. Subsequent studies have identified that KEAP1 is a cytosolic protein that inhibits the NRF2 activity by acting as an adaptor for Cullin-3-based E3 ubiquitin ligase complex [35]. Due to the existence of a large number of cysteine residues, it has been proposed that KEAP1 is a sensor molecule for oxidative stress through Michael reaction and, based on this conjecture, the so-called cysteine code hypothesis was proposed, in which the structural changes of KEAP1 by thiol modifications of redox-sensitive cysteine residues alter and regulate the KEAP1 activity [36]. KEAP1 protein consists of 5 different domains: an amino-terminal region (NTR), a Broad Complex, Tramtrack, and Bric-a-Brac (BTB) domain, an intervening region (IVR), six Kelch/double glycine repeats (DGRs), and a carboxy-terminal region (CTR) (Figure 2) [37]. A number of biophysical and structural analyses have by far provided meaningful insights into how KEAP1 might control the NRF2 stability. Using NMR analysis, it was demonstrated that the peptide harboring the Neh2 domain assumes a rod-like structure and the regions flanked by the ETGE and DLG motifs form an *α*-helix [38]. Seven lysine residues located in the Neh2 domain are all potential polyubiquitination sites by KEAP1 and six of them are aligned on the same side of the *α*-helix. However, to the best of our knowledge, the crystal or NMR structure of full NRF2 protein is not available yet, possibly due to its intrinsic insolubility. On the other hand, biochemical studies have demonstrated that KEAP1 employs the DGR region to recognize two primary sequences on NRF2, for example, the ETGE and DLG motifs, both of which are located in the Neh2 domain of NRF2. Crystal structure studies revealed that KEAP1 DC (DGR + CTR) domain forms a barrel structure composed of six *β*-propellers and the ETGE or DLG peptides fit into the bottom of the DC barrel structure. Using a single particle electron microscopy, Tong and colleagues have demonstrated that the overall KEAP1 dimers assume a cherry-bob-like structure [39], in which two round globules are connected with a stem-like structure and each globular structure is a rounded cylinder with a narrow penetrating tunnel. Because the binding affinity of the ETGE motif to KEAP1 is much higher than that of the DLG motif to KEAP1 as demonstrated by the isothermal calorimetry (ITC), the so-called hinge and latch model was proposed [40], in which a strong interaction of KEAP1 with the ETGE acts as a hinge and a weak interaction of KEAP1 with DLG motif is regarded as a latch (Figure 3). While the “hinge and latch” model still holds as a primary model that accounts for the KEAP1 and NRF2 interaction, alternative or disruptive models explaining the NRF2 and KEAP1 interaction and the resulting activity were also provided by employing different experiment approaches [41].

## 4. The Janus Faces of NRF2: Good or Evil?

It is generally accepted that the induction of NRF2-dependent gene expression contributes to the detoxification of intracellular ROS, thereby alleviating the oxidative damage in organisms. This assumption is well supported by the observation that NRF2 knock-out mice were highly susceptible to oxidative stress-mediated injuries or carcinogenesis, compared with wild-type littermates [42]. Hence, it is plausible to assume that enhancing the activity of NRF2 would be beneficial to attenuate or block the progression of proinflammatory diseases. In line with this idea, a number of chemopreventive agents, including sulforaphane, curcumin, resveratrol, and a synthetic terpenoid, 2-cyano-3,12-dioxooleana-1,9(11)-dien-28-oic acid (CDDO-Me), unequivocally resulted in the attenuation of proinflammatory diseases through an induction of NRF2-dependent phase II cytoprotective enzymes in a variety of experimental animal models [43–46]. Notably, dimethyl fumarate (DMF), a strong inducer of NRF2, was recently approved by the Food and Drug Administration (FDA) with a brand name, Tecfidera, for treatment of recurrent multiple sclerosis (MS) patients [47]. This fact validates the feasibility of KEAP1/NRF2 signaling pathway as a drug target. Although diverse mechanisms might be involved, it is speculated that the induction of phase II cytoprotective enzymes by NRF2 chemical inducers occurs, at least in part, by modulating the activities of intracellular signaling kinases. This assumption is well supported by many previous experimental observations that genetic ablation or treatment of pharmacological kinase inhibitors significantly affected the NRF2/ARE-dependent gene expression [48]. While it is certain that multiple intracellular signaling kinase cascades such as PKR-like endoplasmic reticulum kinase (PERK), phosphatidylinositol 3′-kinase (PI3K), and protein kinase C (PKC) are involved, the exact mechanisms underlying how these individual kinases are orchestrated to regulate the NRF2/ARE-dependent gene expression are relatively uncertain. Therefore, additional studies elucidating direct NRF2 kinases and their exact phosphorylation residues in NRF2 are necessary. By now, only two direct NRF2 kinases are reported: PKC delta (PKC*δ*) is known to phosphorylate NRF2 at serine 40 to activate the ARE-dependent gene expression, and Fyn kinase can phosphorylate NRF2 at tyrosine 568 to suppress the ARE-dependent gene expression. However, whether and, if so, how NRF2 phosphorylation contributes to the NRF2 stability, for example, polyubiquitination, are also unclear.

On the other hand, recent studies have indicated that cancer cells hijack the ability of NRF2 to survive under the oxidative or electrophilic conditions. This conjecture is supported by epidemiological observations that KEAP1 and NRF2 are abundantly mutated in various types of human cancer [49–51]. In addition, recent studies have established a role for NRF2 in modulating anabolic pathways to deal with metabolic demands of cancer cell growth and proliferation. Finally, an increased NRF2 activity is positively correlated with a poor prognosis and chemotherapeutic resistance [52]. It is known that multiple KEAP1 missense mutations occur in human lung adenocarcinoma and they are not limited in certain domains but widely distributed throughout KEAP1 [33]. No matter where KEAP1 mutations occur, they seem to promote the overall stability and/or nuclear translocation of NRF2, thereby contributing to the NRF2/ARE-dependent gene activation. On the other hand, NRF2 mutations were observed in patients in lung, esophagus, skin, and head and neck cancers [53, 54]. Unlike KEAP1, most NRF2 mutations were confined to the ETGD and DLG motifs, providing an indirect support for the hinge and latch hypothesis in the clinical setting. Another interesting aspect is that the occurrence of KEAP1 and NRF2 mutations is mutually exclusive in cancer patients, suggesting that targeting either KEAP1 or NRF2 is sufficient to activate ARE-dependent gene expression in cancer. In addition, recent studies have identified that some proteins bear analogous peptide sequences with the ETGE or DLG motif, which helps them to interfere with the molecular interaction between NRF2 and KEAP1. For example, Chen et al. have demonstrated that p21, a target of p53-mediated cell cycle and apoptosis, can associate with the DLG motif in NRF2 and increase the NRF2 level, resulting in the inhibition of KEAP1 and NRF2 interaction [55]. In addition, Komatsu et al. [56] have demonstrated that p62, a polyubiquitination binding protein that targets substrates for autophagy, contains the STGE motif and it stabilizes NRF2 by inhibiting the polyubiquitination of NRF2 by KEAP1. Together, the involvement of p21 and p62 in the regulation of KEAP1/NRF2 lends a good support for the assumption that modulating the NRF2/ARE signaling pathway is critical in executing the cell-cycle arrest or autophagy in cancer.

Cancer-preventive activity of NRF2 has been well demonstrated in experimental settings, not only by showing that enhanced NRF2 activity results in inhibition of carcinogenesis through its cytoprotective effects, but also by showing that impaired function of NRF2 through genetic deletion of NRF2 increased a susceptibility to cancer formation [57]. Consistent with this view a number of chemopreventive agents, such as sulforaphane, curcumin, CDDO-Me, and DMF, are effective in treating diverse proinflammatory diseases, via activation of NRF2 and a subsequent induction of antioxidative and cytoprotective enzymes.

On the other caveat, NRF2 is also considered as oncogenic and the results of several studies support this view. DeNicola et al. [58] showed that NRF2 might play a role in oncogenesis through elegant genetic animal studies. NRF2 can upregulate antiapoptotic proteins such as Bcl-2 and Bcl-xL [59] and the rate of glycolysis to promote cell proliferation, thereby contributing to cancer cell survival [60]. In the analysis of clinical samples, it was found that gain-of-function mutations in NRF2 exist in carcinomas of esophagus, skin, and larynx, while loss-of-function mutations in KEAP1 are observed in carcinomas of lung, gall bladder, ovary, breast, liver, and stomach [51]. Therefore it can be surmised that continuous activation and accumulation of NRF2 due to perturbed regulation and mutation will lead to chemotherapeutic resistance [61]. The double faced function of NRF2 in different contexts indicates that NRF2 can be both antitumorigenic and protumorigenic.

## 5. Concluding Remarks

By now, we have discussed the molecular mechanisms underlying the detoxification of intracellular ROS and how H_2_O_2_ participates in the activation of signal transduction and contributes to cell proliferation and growth. We have also provided structural insights demonstrating how KEAP1 regulates the NRF2 stability and coordinates the adaptive defensive responses against oxidative stress. Finally, we have provided an evidence that an increased NRF2 activity in normal cells is protective and beneficial against oxidative stress, but cancer cells harness the ability of NRF2 to survive under stress conditions. Due to this contradictory role of NRF2 in cancer, it is important to determine whether NRF2 genotype could be beneficial or detrimental in the development of other chronic diseases, considering a broader implication of oxidative stress in the pathogenesis of numerous human diseases. The existence of various genetic tools, including NRF2 knock-out mice model, can help to address this issue.
